# Uremic Toxins and Blood Purification: A Review of Current Evidence and Future Perspectives

**DOI:** 10.3390/toxins13040246

**Published:** 2021-03-30

**Authors:** Stefania Magnani, Mauro Atti

**Affiliations:** Aferetica S.r.l, Via Spartaco 10, 40138 Bologna (BO), Italy; mauro.atti@aferetica.com

**Keywords:** uremic toxins, protein-bound toxins, blood purification, hemoperfusion, adsorption

## Abstract

Accumulation of uremic toxins represents one of the major contributors to the rapid progression of chronic kidney disease (CKD), especially in patients with end-stage renal disease that are undergoing dialysis treatment. In particular, protein-bound uremic toxins (PBUTs) seem to have an important key pathophysiologic role in CKD, inducing various cardiovascular complications. The removal of uremic toxins from the blood with dialytic techniques represents a proved approach to limit the CKD-related complications. However, conventional dialysis mainly focuses on the removal of water-soluble compounds of low and middle molecular weight, whereas PBTUs are strongly protein-bound, thus not efficiently eliminated. Therefore, over the years, dialysis techniques have been adapted by improving membranes structures or using combined strategies to maximize PBTUs removal and eventually prevent CKD-related complications. Recent findings showed that adsorption-based extracorporeal techniques, in addition to conventional dialysis treatment, may effectively adsorb a significant amount of PBTUs during the course of the sessions. This review is focused on the analysis of the current state of the art for blood purification strategies in order to highlight their potentialities and limits and identify the most feasible solution to improve toxins removal effectiveness, exploring possible future strategies and applications, such as the study of a synergic approach by reducing PBTUs production and increasing their blood clearance.

## 1. Introduction

Chronic kidney disease (CKD) is characterized by a progressive degradation of glomerular filtration, which leads to the loss of the kidneys’ normal capability to remove potentially toxic molecules from the blood circulation through the urine, resulting in their accumulation [1].

These molecules are called uremic retention solutes, and if they are biologically or chemically active, they are called uremic toxins [1]. The accumulation of these toxins has negative effects on the physiological functions, resulting in a gradual endogenous intoxication and in a progressive deterioration of the clinical conditions [1,2].

The European Working Group on Uremic Toxins (EUTox) has identified more than 90 uremic toxins [2], that are classified in three categories according to their molecular weight and chemical characteristics [1], which affect their removal and, consequently, the appropriate extracorporeal purification strategy [3].

The first category includes free water-soluble low molecular weight molecules (< 0.5 kDa), such as creatinine and urea, which are easily and efficiently removed by conventional dialysis [1,2,3].

Middle molecular weight molecules (0.5–60 kDa), including peptides and proteins, are part of the second category and prototypes are β2-microglobulin and α1-macroglobulin. These compounds, due to their molecular weight, can only be removed by dialysis treatment performed with dedicated dialyzer characterized by larger pores on the membrane surface [4].

The last category is represented by strongly protein-bound uremic toxins (PBUTs), which are relatively low molecular weight solutes binding to large carrier proteins, mainly albumin [1,2,3,5,6]. The principal prototypes are indoxyl sulfate (IS) and p-cresyl sulfate (PCS), presenting a protein bound fraction higher than 90% [6]. This high binding affinity for albumin impairs the PBUTs clearance with conventional haemodialysis treatments, even with high-flux methods [7,8]. Considering the IS and PCS example (protein-bound and free molecule), their reduction rates by conventional haemodialysis are only 31.8% and 29.1%, respectively [7], very limited in light of their high toxic activity towards the body [8].

Several studies demonstrated the correlation between PCS, particularly the free form [9], and a higher mortality due to cardiovascular diseases in patients with CKD and end-stage renal disease (ESRD) [10]. Similarly, such direct associations also with IS are reported in the literature [11]. Indeed, accumulation of these solutes represent a crucial point in CKD since many toxins in the PBUTs group play an important pathophysiologic role in CKD, accelerating its progression and primarily impacting the cardiovascular system, particularly in patients with ESRD and that are undergoing dialysis treatment [3,8,9,10,11].

Therefore, effective removal of PBTUs remains one of the major challenges in the dialytic field and many techniques, membranes and combined strategies have been studied to maximize their removal and eventually prevent CKD-related complications. Bearing this in mind, this narrative review is focused on the analysis of the current state of the art for blood purification strategies in order to highlight their potentialities and limits and identify the most feasible solution to improve toxins removal effectiveness, exploring possible future strategies and applications.

## 2. Extracorporeal Blood Purification

### 2.1. Conventional Dialysis

Conventional haemodialysis (HD) represents the main technique adopted for the reduction of high concentrations of uremic toxins from the blood [12]. Over the years, dialytic treatments have improved the effective removal of many uremic toxins, mainly small water-soluble compounds and middle molecules, but traditional methods have limited effects in providing an adequate PBTUs removal and seem to have no major impact in preventing or slowing down cardiovascular damages [12].

**Figure 1:**
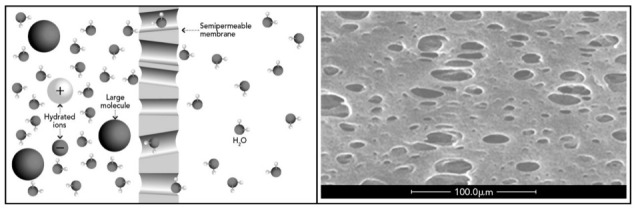
Mechanism of a semipermeable membrane (**left**) and porous surface of a hollow fiber membrane (**right**).

The limited removal effectiveness is linked to the high ratio of distribution volumes and the strong protein-bond of various PBTUs molecules, which impair their removal during HD as a direct consequence of the characteristics of the dialyzer membranes [13]. The mechanism of action is based on the physical transportation of solutes through a semipermeable membrane via the principles of diffusion and/or convection. Solute transport across semi-permeable membranes takes place through the pores that characterize the surface, and their size and distribution morphology influence the purification behaviour [13,14], as shown in Figure 1. The pores’ cut-off is variable but it always keeps as target the albumin molecular weight (66.5 kDa) to set the membrane permeability, in order not to cause hypoalbuminemia or other deleterious nutritional consequences [15].

Consequently, middle molecular weight molecules and PBTUs are poorly removed with HD treatment compared to free water-soluble low molecular weight molecules, due to their nature or bond with albumin [12].

Martinez and co-workers [16] showed the limited PBTUs clearance with HD analysing the behaviour of PCS and indican (IS), compared to urea. The free fractions of both PBTUs declined rapidly during the course of the treatment whereas solute reduction ratios were quite different from those for urea: 20% for PCS, 30% for indican, and 69% for urea. These results reflected that the protein binding and high-volume ratio of the PBTUs studied limited their clearance with HD and low-flux (LF) filters. Another study observed a comparable behaviour for some PBTUs, including IS and PCS, which could not be removed efficiently by HD and presented low reduction rates (< 35%) [7].

Over the years, improvements in reduction of small and middle toxins could be obtained by varying different factors, as the number of HD sessions and the treatment time extension [12,17]. Basile et al. [17] studied the role of the HD treatment time in the removal of uremic toxins, observing that this factor is extremely important for small and middle molecules, but not for protein-bound solutes.

The real difference in middle molecular weight molecules removal has been made with the use of hemodiafiltration (HDF) and the development of high-flux membranes (HF) that could improve the effectiveness due to the increase of convection and the larger pore size and distribution [3,12,18,19].

Indeed, HF membranes have been developed increasing their permeability by enlarging the pore size and raising the ultrafiltration coefficient than the low-flux filters normally used. In this way, they are more efficient in removing medium molecules. However, they present the disadvantage of potential albumin and nutrient losses during the treatment, and the consequent need of their reinfusion [15]. Their ability has been widely demonstrated in literature [18,19], nevertheless the superiority is pronounced regarding middle molecules, but limited data is available referring to PBTUs [3,12].

A recent work of van Gelter et al. [20] investigated whether HDF is able to significantly affect the plasma concentrations of PBUTs, and whether PBUTs are related to the outcome, comparing this technique with LF dialysis. The results showed that treatment with HDF for 6 months did not consistently decrease total PBUT plasma concentrations compared with HD. Moreover, the authors also speculated about the role of HDF in the small solute clearance, including the free PBUTs fraction, considering the absence of a reduction in pre-dialysis PBUT plasma concentrations. This may be explained through the consideration that the free PBTUs fraction has a low molecular weight therefore is very efficiently removed by diffusion with conventional HD.

The limited effects of HDF and HF membranes on PBTUs removal was also demonstrated by Lesaffer and co-workers who conducted a comparative study between HF and LF membranes (cellulose triacetate and polysulfone) used in HD treatments in order to evaluate the PBTUs removal [13]. The results showed that HF membranes might eliminate protein-bound solutes. However, no significant differences were observed between the HF membranes and the LF ones (IS 34.4% vs 36.3% and p-cresol 32% vs 28.1%, respectively). These results were similar to the ones obtained in other studies [7,21], where especially IS removal was elevated even if less than 35% and differences in concentration between HF and LF were relatively small.

Hence, HF seemed not to allow a superior PBTUs removal than LF filters as well as the several modifications proposed in dialytic strategies could not increase the reduction above 35–40% and, more importantly, the observed reductions did not show a clinical significance in CKD complications, still representing a crucial point to demonstrate [1,2,3,12,13,19,20,21].

### 2.2. The Evolution towards Adsorption-Based Techniques

Additional therapeutic strategies have been explored with the aim of improving the PBTUs removal with HD in order to break the ‘albumin wall’ and adsorption-based techniques seemed the most promising approach to this purpose [3,22].

The idea of using a sorbent material to increase the dialysis effectiveness is not recently discovered, having been first used by Muirhead and Reid in 1948, and later by Yatzidis in 1964 in hemoperfusion (HP) treatments to eliminate uremic toxins [23]. However, due to various adverse effects, including poor biocompatibility, significant platelet loss, and haemolysis, sorbents have seen limited use in clinical practice. In the late 1990s, improvements in the materials and production processes resulted in the development of new potential sorbents and interest in their use in extracorporeal purification systems grew [23]. Since then, these techniques have been widely used in different clinical applications, as drug intoxication, sepsis for cytokine removal, or liver dysfunction with the focus on bilirubin [23,24,25,26].

**Figure 2:**
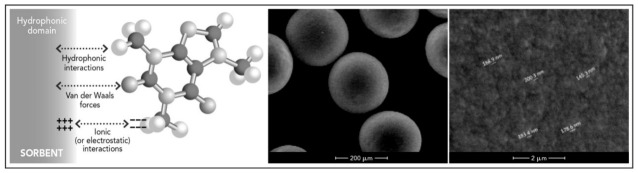
Mechanism of adsorption (**left**), polymeric beads (**center**) and structure of the porous polymeric resin surface (**right**).

Biomedical sorbents are generally hydrophobic solid materials, mainly charcoal or porous polymeric resins, that work directly on whole blood in HP, or on plasma, which must be extracted using an appropriate equipment, on the basis of their bio- and hemo-biocompatibility [22,23,24,25]. The mechanism of action is based on adsorption, a chemical-physical phenomenon that consists in the interaction of one molecule with the surface of the sorbent material mainly by establishing hydrophobic interactions, electrostatic or ionic attraction, and van der Waals forces [22]. The principle and structure of an adsorption material is shown in Figure 2.

Molecule adsorption takes place inside the porous structure of variable size and diameter that characterizes the surface, limiting therefore the passage of molecules with a molecular weight higher than the pore size. In accordance with the IUPAC recommendations, pores are divided on the basis of their inlet size: micropores (<2 nm); mesopores (2–50 nm); and macropores (>50 nm) [22]. For clinical purposes, mesopores are mainly relevant for allowing the adsorption of molecules of medium molecular weight without affecting albumin concentrations.

HP alone does not provide fluid balance and small uremic toxins removal. Therefore, the clinical therapeutic strategy should be the optimization of HD techniques by integrating the sorbent materials.

The first attempt in this direction is represented by the hemodiafiltration with endogenous infusion (HFR) technique that combines at the same time convection, diffusion, and adsorption as purification mechanisms [27,28]. The principle is to use a double stage filter that consists of a high permeability filter in the first convective stage and a low flux filter in the second diffusive stage. The first stage allows the separation of ultrafiltrate from the blood that is addressed in a second circuit through a sorbent resin cartridge able to adsorb several uremic toxins and cytokines, but not albumin [29]. The purified ultrafiltration is then reinfused into the blood and passes through the second stage that performs traditional HD.

To increase the performance of this technique, researchers have developed the SUPRA HFR [30,31,32] using super high flux membrane in the convective stage to obtain an ultrafiltrate richer of albumin and, consequently, PBTUs. This makes it possible to perform without albumin loss considering that the sorbent resin shows the ability not to retain albumin [29]. Results showed that HFR may improve uremic protein-bound toxin removal, inflammatory state, endothelial damage, and oxidative stress [33] but few supporting data are available for the specific removal of PCS and IS [34]. A recent comparative study showed that plasma total p-cresol decreased by about 53% after HFR, and only 37% after HD [34], highlighting the potential of the technique.

**Figure 3:**
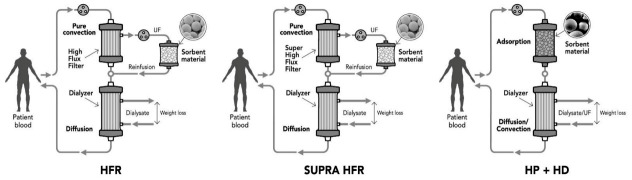
Evolution of the adsorption-based techniques for PBTUs removal during the years. HFR: hemodiafiltration with endogenous infusion; SUPRA HFR: hemodiafiltration with ultrafiltrate regeneration by resin adsorption; HP: hemoperfusion; HD: conventional dialysis; UF: ultrafiltrate.

Technological evolution, in terms of materials, biocompatibility, and production processes, has led to more feasible solutions with the same results through the integration of the sorbent material directly in the current HD systems without any ultrafiltrate or plasma separation. A representation of the technical configuration solutions over time is represented in Figure 3.

Regarding the available sorbent materials, activated carbon has been one of the most studied sorbent materials in the past, although nowadays it is used only rarely and in limited application areas such as the treatment of drug intoxications [22,25,26]. Meyer and co-workers [35] firstly verified in-vitro the capacity of sorbent charcoal to increase protein-bound solute clearances, obtaining twice the removal compared to HD alone while not affecting the unbound solutes. Similarly, a recent study confirmed previous in-vitro findings by performing an in-vitro experiment to assess the efficacy of activated carbon through direct HP with both bovine blood and HD patients’ blood [36]. The PTBUs levels decreased in bovine blood in a dose-dependent manner reaching an IS reduction rate (RR) of 94.5% after 60-min circulation. IS and PCS were drastically adsorbed from the HD patients’ blood taken pre- and post-session, leading the authors to conclude that carbon sorbents may represent a promising strategy for removing circulating PTBUs [36]. Another recent work aimed to better investigate the carbon-based sorbents by manipulating the pore structure to increase the removal of uremic toxins and other molecules, such as cytokines. The sorbent used (CMK-3 type) presented two distinct pore domains: micropores and mesopores [37]. The results showed a high adsorption capacity toward small toxins, such as creatinine, and PTBUs, in particular IS and hippuric acid. Remarkably, the total plasma protein did not decrease after the 4-h experiment and the PTBUs removal seemed to be related to the protein binding: the higher the concentration of the free fraction, the higher the removal [37].

Apart from charcoal, many resin sorbents have been experimented, mainly cellulosic or polymeric [38,39,40,41]. Hexadecyl-chains immobilized in porous cellulose beads in a column was tested in-vitro and in combination with a conventional HD treatment [39]. The adsorption of IS in-vitro was dose and time-dependent reaching 54.9% of removal in 4 h and, on the other hand, albumin did not decrease. In the clinical setting, the column decreased significantly the serum level of free IS by 34,4%, but the total IS levels did not change. The authors hypothesized that the adsorption occurred through the electrostatic interaction between hexadecyl and the amino groups in free uremic toxins, thus more effective materials should be designed [39].

Another recent work by Rocchetti and co-workers [40] tested firstly in-vitro two different resins, including a sorbent based on divinylbenzene coated with a highly biocompatible polymer and the previously cited cellulose with hexadecyl-chains. Regarding the first sorbent, serum total levels of IS and PCS removal after 6 h were 53.7% and 56%, respectively, whereas it reached only 25% and 31.7%, respectively, with the second one. Albumin removal was limited on average within 10% with both sorbent materials. The divinylbenzene resin was also tested in a clinical trial in combination with the HD treatment. The authors observed a discrepancy between in vivo and in-vitro data: the resin adsorbed significantly only IS plasma levels [40], similarly to previous results [39]. At the base of the rationale of this study, the divinylbenzene sorbent had demonstrated the adsorption ability of hydrophobic compounds presenting the same chemical structure and strong protein binding with albumin [41,42] as the PTBUs, in particular IS and PCS.

The analysis of the available results [35,36,37,38,39,40] represents a fundamental step toward the evaluation of the useful information in the development or optimization of strategies to increase the PBTUs’ effectiveness removal during HD treatments. Many speculations could be made in order to understand the different behaviours in-vitro and in vivo, which are probably related to the standardized setting used in the in-vitro studies whereas many factors could interfere in the in-vivo setting, including proteins other than albumin [39,40].

All the results showed a significant reduction in free serum levels of PBTUs instead of total ones [37,38,39,40]. Deltombe explained the difficulty in reducing total levels of PBTUs with the hypothesis that the equilibrium between free and bound is continuously disturbed [43]. First the free fraction is removed, causing a disequilibrium with the bound fraction and the extravascular spaces. Subsequently, the bound fraction is slowly released, and the equilibrium is restored. This hypothesis might explain also the increase in binding percentage observed in some studies and the possible influence in restoring the balance of some factors, such as pH [40,43].

This point still represents a topic to investigate since many speculations have been made about the real adsorption mechanism regarding protein-bound hydrophobic molecules and the possible reversible nature of the bond [41,42,43,44].

### 2.3. Future Perspectives

To date, many potential strategies have been investigated without obtaining significant improvements in the PBTUs removal and, consequently, showing a clinical significance in CKD complications. A summary of the main preclinical and clinical studies that were analysed before is reported in Table 1.

Definitely, an adsorption-based technique seems the most suitable strategy for the desired purposes, but many factors should be understood and improved to increase the long-term effectiveness, guaranteeing at the same time simplicity and applicability in ordinary HD treatments.

However, evidence showed that these techniques alone are not sufficient, even presenting higher removal ability than HD [34,35,36,37,38,39,40], therefore, it would be important to act on two fronts.

Firstly, research should be focused on the improvement of the sorbent materials with technical characteristics able to optimize the affinity against PBTUs. As previously discussed, porosity and pore size represent the key properties that could affect the affinity of a compound [22,23,24]. In a study of Harm et al. [45], the optimal pore size for toxin removal was investigated with the finding that adsorbents with 30–40 nm pores are the best choice for the removal of albumin-bound toxins in the case of liver failure, but this is applicable to other fields with similar molecules [45]. Together with these properties, the active adsorption surface and material hydrophobicity are also important to increase the removal in the first hours and the affinity against the hydrophobic PBTUs molecules.

According to the evidence, the divinylbenzene sorbent coated with polyvinylpyrrolidone had demonstrated the major adsorption ability regarding PBTUs [40] and other similar protein-bound compounds [41,42], without causing a significant drop in albumin [40,41,42], due to the fact that albumin interacts mostly through hydrophilic bonds, whereas styrene copolymers interact mostly through hydrophobic ones [42]. The pores of this sorbent materials are generally 5-15 nm; consequently, a first step could be the manipulation of the pore structure in the production process reaching 30-40 nm.

The second research area should concentrate on the development of combined strategies by acting on the PBTUs production and their clearance from circulating plasma. In general, the sole removal of a toxin from the circulation is not sufficient if there is a continuous production by the organism. Consequently, acting on two levels seems a reasonable and valuable strategy to maximise the PBTUs removal and obtain long-term clinical effects. Rocchetti and co-workers have tried this approach in their clinical trial [40]. Patients were treated with a symbiotic to reduce the PBTUs production by gut microbiota modulation at colonic levels and an adsorption-based HD treatment to reduce PBTUs serum levels. The synergy of the treatments decreased IS and PCS both at pre- and post-HD levels in the treatment group and not in the placebo one [40], representing the first proof of concept of the potential synergic strategy.

A future direction is to further explore this synergic strategy by optimizing the sorbent technology, the HD treatments setting, and the PBTUs production, without significantly affecting CKD patients’ clinical routine.

## 3. Conclusions

Over the years, progresses in HD treatments have increased the effectiveness in reducing small and middle weight molecules involved in CKD. A challenge target has always been represented by PBTUs and their involvement in CKD-related cardiovascular and systemic complications is widely demonstrated. For this reason, many strategies have been investigated, including modifying the conventional HD setting, such as the treatment duration and the number of sessions, introducing HF membranes and, lastly, adding adsorption-based therapies, in particular HFR and HP integrated directly in the current HD systems. Nonetheless, few supporting scientific experiences in the literature are available with conclusive results, especially showing the control of CKD complications. Certainly, adsorption-based techniques showed better clearance for middle weight molecules and PBTUs but alone they are not sufficient.

Future research should be addressed on the evaluation of a feasible synergic approach by reducing PBTUs production in the upstream and increasing their clearance in the downstream. At the same time, the impact of their removal on clinical outcomes and mortality should be further investigated.

## Figures and Tables

**Table 1:** Summary of the main pre-clinical and clinical studies regarding the use of blood purification strategies to improve protein-bound toxins (PBTUs) removal during the course of the years.

Authors	Population	Extracorporeal Technique	Results	References
Martinez AW. et al.	5 chronic HD patients	Conventional HD	PCS and Indican were poorly removed by HD.	[16]
Itoh Y. et al.	45 HD patients	Conventional HD	IS, PCS and CMPF could not be removed efficiently by HD due to their high protein-binding ratios.	[7]
Basile C. et al.	11 anuric HD patients	Conventional HD with an extended treatment time	Small and middle molecules were removed more adequately when performing a prolonged HD, whereas no statistically significant difference was observed for PBTUs.	[17]
Krieter DH. et al.	8 HD patients	Conventional HD vs HDF	The decreases of PBTUs were comparable between HD and HDF treatment forms.	[18]
Lesaffer G. et al.	10 chronic HD patients	High-Flux dialysis vs HD	High-Flux membranes did not appear to be superior in removing PBTUs compared to HD.	[13]
Van Gelder MK. et al.	80 HD patients	Online HDF vs LF HD	The treatment with HDF for 6 months did not consistently decrease total PBUTs plasma concentrations compared with HD.	[20]
Monari E. et al.	14 HD patients	HFR vs Supra-HFR	Results indicated that Supra-HFR showed higher efficiency in removal of middle molecules related to uremic syndrome.	[32]
Esquivias-Motta E. et al.	17 HD patients	HFR vs online-HDF	HFR was associated with greater IS removal and appeared to improve PBTUs removal, inflammatory and endothelial status, and oxidative stress.	[33]
Riccio E. et al.	12 inflamed chronic HD patients	Supra-HFR	HFR-Supra cartridge showed the ability to decrease total PCS and IL-6 in the ultrafiltrate while only the PCS levels were lowered in the plasma.	[34]
Meyer TW. et al.	In-vitro experiment	Activated charcoal + HD	The addition of sorbents to HD could increase the clearance of PBTUs, obtaining twice the removal compared to HD alone.	[35]
Yamamoto S. et al.	In-vitro experiment	Activated charcoal in direct HP	Activated charcoal effectively adsorbed blood PBTUs in vitro.	[36]
Pavlenko D. et al.	In-vitro experiment	Manipulated carbon-based sorbents	The results showed a high adsorption capacity toward small toxins, such as creatinine, and PTBUs, in particular IS and hippuric acid, but the total PBTUs levels did not decrease after the 4-h experiment.	[37]
Yamamoto S. et al.	In-vitro experiment + 17 HD patients	Hexadecyl-immobilized in porous cellulose beads + HD	The adsorption of IS in-vitro reached 54.9% in 4h while in-vivo the column decreased significantly the serum level of free IS by 34,4%, but the total IS levels did not change.	[39]
Rocchetti MT. et al.	In-vitro experiment + 11 HD patients	Divinylbenzene vs cellulose sorbents + HD + symbiotic treatment	In-vitro data showed that divinylbenzene sorbent was more effective in adsorbing IS and PCS after 6h perfusion. The combination of symbiotic treatment with divinilbenzene sorbent HD showed the decrease of IS and PCS both at pre- and post-HD levels.	[40]

HD: conventional haemodialysis; HDF: haemodiafiltration; HP: hemoperfusion; PCS: p-cresil sulphate; IS: indoxyl sulphate; CMPF: 3-carboxy-4-methyl-5-propyl-2-furanpropionic acid; PBTUs: protein-bound uremic toxins; HFR: Hemodiafiltration with endogenous reinfusion; HFR SUPRA: hemodiafiltration with ultrafiltrate regeneration by resin adsorption.

## Data Availability

Not applicable.
